# Evaluating Biocompatibility: From Classical Techniques to State-of-the-Art Functional Proteomics

**DOI:** 10.3390/nano15131032

**Published:** 2025-07-03

**Authors:** Ana Nuño-Soriano, Carlota Arias-Hidalgo, Enrique Montalvillo, Rafael Góngora, Ángela-Patricia Hernández, Pablo Juanes-Velasco, Manuel Fuentes

**Affiliations:** 1 Translational and Clinical Research Program, Cytometry Service, NUCLEUS and Department of Medicine, Cancer Center Research (IBMSS-CSIC), University of Salamanca, 37008 Salamanca, Spain; ananunsor@usal.es (A.N.-S.); carlotaariashidalgo@usal.es (C.A.-H.); emontalvillo@usal.es (E.M.); rgongora@usal.es (R.G.); pablojuanesvelasco@usal.es (P.J.-V.); 2 Proteomics Unit-IBSAL, Institute of Biomedical Research of Salamanca, University of Salamanca (IBSAL-USAL), 37007 Salamanca, Spain; 3 Department of Pharmaceutical Sciences: Organic Chemistry, Faculty of Pharmacy, University of Salamanca, CIETUS, IBSAL, 37007 Salamanca, Spain

**Keywords:** biocompatibility, biomaterials, nanomedicines, proteomics, toxicity, functionality, nanoscale

## Abstract

Biocompatibility remains a central issue for introducing biomaterials and nanomedicines into the clinic, requiring safety, functionality, toxicity prevention, and the control of foreign body reactions. Therefore, it is necessary to evaluate multiple biomaterial parameters and molecular interactions affecting cell functions, like apoptosis, adhesion, proliferation, or spreading, as well as intracellular signals and cellular microenvironment status. Although conventional well-established in vitro techniques are helpful at the first stages of bio and nanomaterials development, high-throughput techniques expand the screening and designing possibilities. This review presents high-throughput functional proteomics approaches, focused on protein microarrays and mass spectrometry techniques, for the evaluation of biocompatibility in the new era of biomedicine.

## 1. Introduction

**Figure 1:**
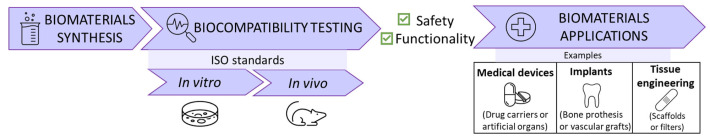
Biomaterials development process: synthesis, in vitro and in vivo testing, and multiple clinical applications.

Biomaterials have improved quality of life and contributed to enhancing medical approaches (such as diagnosis, prognosis, therapies, etc.) thanks to the continuous design and development of novel technologies [1]. There is great diversity in designed biomaterials, as they use a huge variety of physical and chemical conformations, such as polymers, ceramics, hydrogels, silicones, metals, or nanoparticles [2]. Hence, the large number of structures and compositions increases biomaterial variety for biomedical applications (artificial organs, drug carriers, immunomodulatory agents, etc.) [3], implants (dental implants, bone prosthesis, intraocular lenses, vascular grafts, heart valves, etc.) [4], or tissue engineering and regeneration (scaffolds, filters, coating, etc.) [1,2,3,5] (Figure 1). While biomaterials are defined by the application of materials into biological systems, nanomaterials are materials characterized by having at least one dimension in the range of 1 to 100 nm, being in solid state and possessing defined physical boundaries (such as a specific surface area by volume of >6 m^2^/cm^3^) [6]. Although not all nanomaterials are intended for biological use, their large range of dimensions, compositions, and properties expand their potential in medical applications (nanomedicine), such as personalized medicine [7,8].

As biomaterials, it is important to guarantee nanomaterials’ biosafety and evaluate their compatibility [9]. Biocompatibility is the ability of a material to be in contact with the host without causing adverse and/or unexpected effects in a defined timeframe (distinguishing between acute effects observed in the short term and chronic effects in long term) [10]. Then, biocompatibility encompasses the combination of the safety and functionality of a material [11]. Nowadays, there is a growing need for novel materials with surface functionalities that support biological viability [12,13]. For this purpose, biocompatibility assays evaluate toxicity, interactions with the environment and resistance to colonization by extrinsic organisms [14,15] (Figure 1).

Biocompatibility can evaluate biomaterials in the clinical healthcare area, but their wide variety of conformations and formats extends the scope of their applications in different clinical directions. As a result, biomaterials have revolutionized medicine in areas such as odontology [16], cardiology [17], ophthalmology [18], oncology [19], and controlled drug delivery [20,21]. It is necessary to highlight their contribution in regenerative medicine and tissue engineering in different types of tissues such as bone [22], skin [23], pulmonary [24] or nerve tissue [25,26].

Nonetheless, to properly understand biocompatibility assays, it is essential to understand that the compatibility profiles of both biomaterials and nanomaterials are strongly influenced by the response triggered by the immune system (IS) upon contact with the host organism. It is well-described that the mammalian IS recognizes foreign structures/external moieties to elicit a defensive immune response (IR) when it is necessary. Those moieties could be pathogens, or damage-associated molecular patterns (PAMPs and DAMPs, respectively), recognized by pattern recognition receptors (such as Toll-like receptors (TLR)) or organism-specific structures called antigens. When the immune cells recognize them through antigen-specific receptors such as B cell receptors or T cell receptors, an immunological response can be initiated [27,28].

**Figure 2:**
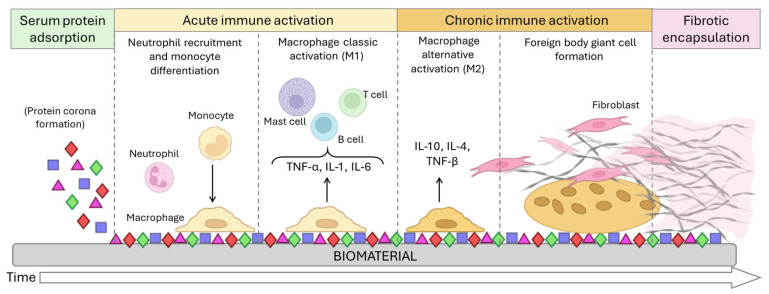
Foreign body response (FBR) process. The FBR begins when serum proteins adhere to the biomaterial surface, forming the protein corona. This corona attracts neutrophils and monocytes, which differentiate into macrophages. Macrophages adhere to the biomaterial, attempt to degrade it, and release pro-inflammatory cytokines that contribute to the recruitment of further immune cells. When the biomaterial is too big for the macrophages to degrade, they fuse into foreign body giant cells. These multinucleated giant cells recruit fibroblasts that accumulate a collagen matrix around the biomaterial for its encapsulation.

As a result of the body’s defence mechanisms, the insertion of any external agent into the body triggers a non-specific inflammatory response called a foreign body reaction (FBR) [29,30]. FBR events are initiated minutes after implantation by non-specific plasma protein adsorption (forming the protein corona; described below that depends on the surface properties of the biomaterial [31,32]. Hours later, acute inflammation driven by mast cells and polymorphonuclear leukocytes occurs, which stimulates the activation of local monocytes/macrophages into their pro-inflammatory state. Multiple cytokines, such as IL-4, IL-13, and TNF-α, and growth factors (vascular endothelial or platelet-derived growth factor) recruit immune cells as B and T lymphocytes that also mediate the IR [30,33]. About 4 to 7 days later, chronic inflammation appears, involving monocyte/macrophage accumulation and macrophage alternative activation (M2) that release other cytokines, such as IL-10, IL-4, or TGF-β. Then, macrophage fusion occurs to form foreign body giant cells and fibroblasts are recruited. Finally, while chronic inflammation declines, collagen encapsulation or fibrosis isolates the implant and vascularization is induced and tissue is repaired [30,31,34,35] (Figure 2).

The chemical, physical, biological, and morphological characteristics of the surface of the inserted material (a special interest in nanomaterials) have an influence on the IS and modulate the IR [36]. Nevertheless, the FBR interferes in the functions of the implants and usually results in the rejection of these devices, becoming the major obstacle for the development of new therapies. Therefore, anti-FBR technologies are the new challenge for improving the clinical outcome and efficacy of several therapies [30,37,38,39]. In Europe, to evaluate toxicity and avoid the FBR, the International Organization for Standardization (ISO) established in vivo and in vitro biocompatibility tests. All of them are collected under the ISO 10993 standard: “Biological Evaluation of Medical Devices” [40]. Toxicity should be studied at different scales, described in ISO 10993, parts 5:2009 (“Tests for In Vitro Cytotoxicity”) [41], 11:2017 (“Tests for Systemic Toxicity”) [42], 20:2006 (“Principles and Methods for Immunotoxicology Testing of Medical Devices”) [43], and 22:2017 for nanomaterials biological evaluation [44] (Figure 1).

The IR is mediated and regulated by one of the main functional biomolecules of the organism, the proteins, which also compose the main patterns for antigen recognition. In this framework, exploring the cell’s response to foreign structures at the proteomic level could be crucial to decipher any mechanisms involved in the biocompatibility of nanomaterials [8,45]. In general, proteomics is the analysis, identification, quantification and characterization of all protein isoforms contained in a sample (cell, tissue, organ, or organism) including their function, expression, and structure [46,47]. Depending on the areas of study, proteomics can be classified into structural proteomics, differential expression, and functional proteomics. Structural proteomics traces the three-dimensional configuration of proteins within a particular organelle, their location, and interactions. Differential proteomics involves the comparison of protein expression profiles within the entire proteome or specific subproteomes between different samples. Functional proteomics explores the role of protein functions, their intracellular signalling pathways, and the interactions among proteins [48,49].

Overall, proteomics has moved from the conventional Western blot, ELISA assay, and gel electrophoresis-based proteomics to other high-throughput and high-content approaches such as protein microarrays and label-free quantitative mass spectrometry (MS) techniques (data-dependent acquisition (DDA) and data-independent acquisition (DIA)) or targeted proteomics (parallel reaction monitoring (PRM) and selected reaction monitoring (SRM)) [48,50].

Although conventional proteomic methods have improved selectivity and specificity [46,51], one of their main limitations is that they can only evaluate a small number of selected proteins. Conversely, advances in high-throughput technologies—described below in Section 3—have intensely improved the depth of proteome characterization [52,53]. High-throughput proteomic techniques are required to integrate multiple strategies and generate large-scale proteomic datasets [12,54]. Bearing in mind that proteomic tools have contributed to clinical diagnostics, disease progression, drug response monitoring, and biomarker identification for treatment and diagnosis, while expanding knowledge on proteome heterogeneity and its biological implications [55], they are considered a strong approach for deciphering and improving the biocompatibility of nanomaterials.

Considering the aforementioned points, this manuscript examines the primary elements influencing biocompatibility assessment through in vitro tests, covering traditional well-established methods as well as novel approaches. Some examples are also presented to illustrate the impact and relevance of implementing proteomic characterization strategies, with the aim of enhancing biocompatibility evaluation and identifying new opportunities. We also considered it necessary to highlight that these techniques are not exclusive to one cellular process or one particular nanomaterial, but complementary to many other biological characterizations. To facilitate the description of the different methods, they are classified according to those methods that are more conventional (here labelled as “classical techniques”) versus those mainly based on proteomic techniques and/or supported by proteomics approaches.

## 2. Classical Techniques

Biomaterials, including nanomaterials, are designed to mimic the physical and structural properties of cells and tissues, but their insertion can influence key cellular characteristics, including morphology, proliferation, differentiation, migration, and survival [36,56]. Therefore, their toxicity or compatibility depends on multiple factors; among others, these include dose, chemical composition, size, surface, structure, solubility, biodegradability, pharmacokinetics, and biodistribution [57]. Bearing this in mind, testing biomaterial safety is a multidisciplinary area, as it might be expected, which requires the combination of different techniques and methodologies. Although novel developments have broadened the evaluable parameters, conventional tools are still useful and widely employed at early stages of biomaterials development, as they are rapid and cheap and their protocols are well-established [58].

**Figure 3:**
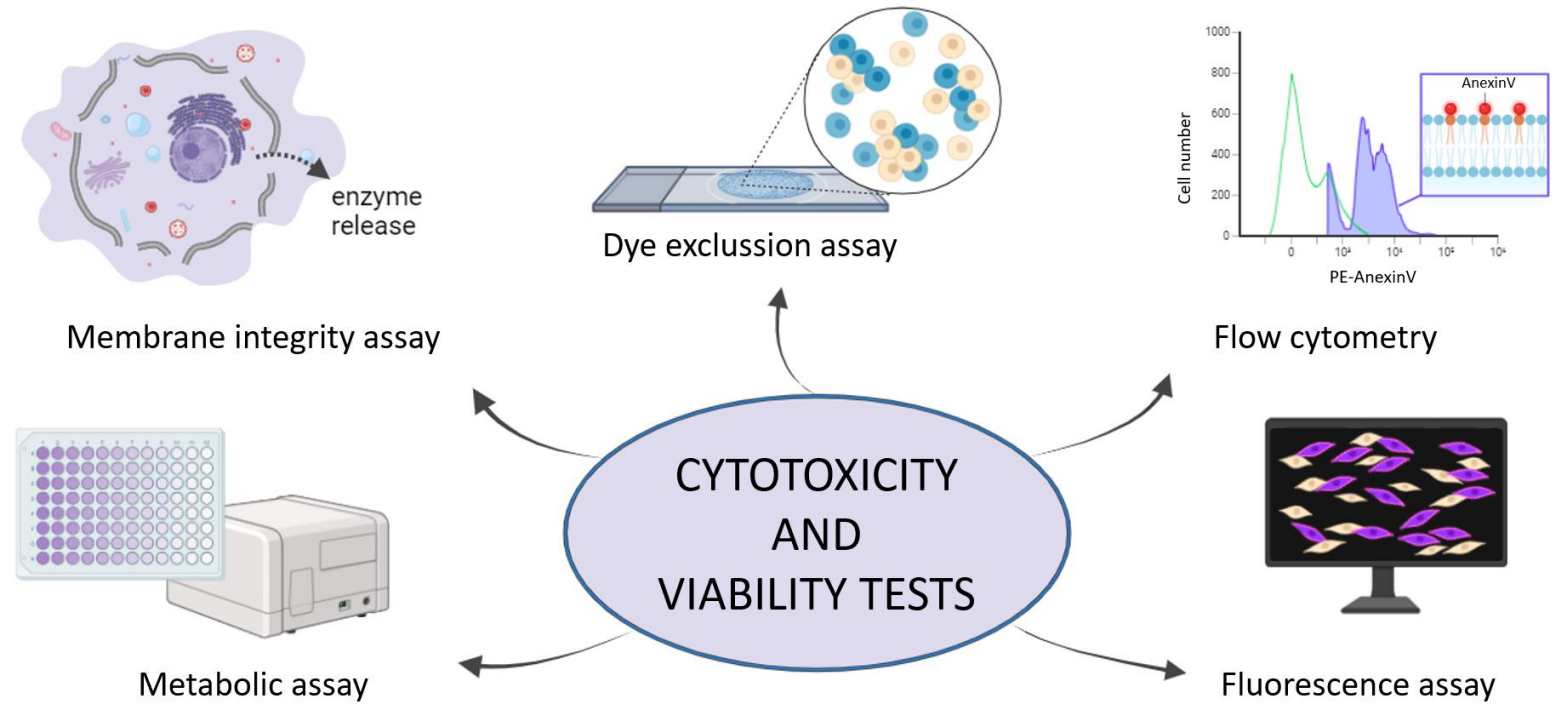
Cytotoxicity and viability test as conventional techniques for studying the biocompatibility.

According to ISO 10993-5:2009 [41], in vitro cytotoxic evaluation is categorized into (a) assessments of cell damage by morphological markers and measurements of cell damage and (b) cell growth and specific features of cellular metabolism [58,59,60]. The classic colorimetric and fluorometric methods include, among others, dye exclusion assays, fluorescence assays, flow cytometry, metabolic assays, and membrane integrity assays [61,62] (Figure 3).

Nonetheless, all of them rely on mammalian cell culture, as sample preparation, to subsequently evaluate the mentioned toxicity. Besides the fact that all of these conventional methods are highly recommended and commonly used for studying cell viability, some limitations still exist, and further developments are required [63] (Table 1).

## 3. Proteomic Approaches for Biocompatibility Evaluation

As previously introduced, novel proteomic approaches open a new window of exploration into multiple parameters to optimize the development of biomaterials, including nanomaterials. Nevertheless, it is necessary to provide a background on the proteomic methods and strategies included in high-throughput approaches. Two main proteomic strategies can be distinguished: microarrays and MS.

**Figure 4:**
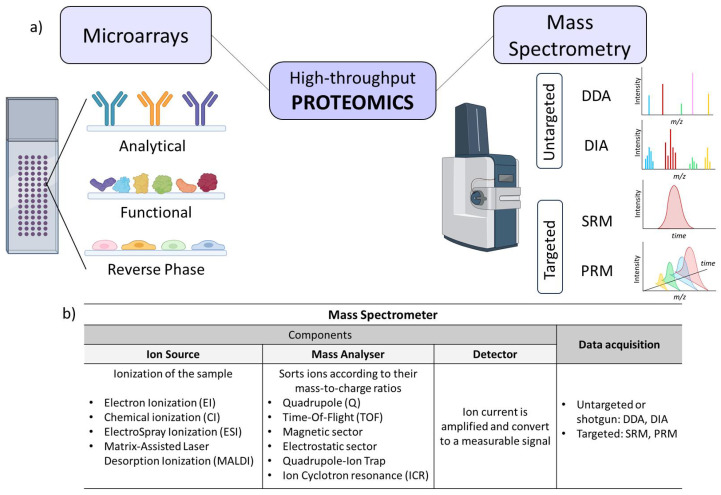
(**a**) High-throughput functional proteomics approaches based on microarrays (represented are the three types of arrays depending on their content) and mass spectrometry (represented are four data acquisition modes and their representative spectrums). (**b**) Overview of the different mass spectrometer component functions, types, and data acquisition.

On the one hand, MS can measure unmodified proteins and post-translational modifications (both simultaneously) within its detection range with high sensitivity [89,90,91]. Nowadays, in MS-based proteomics, it is common to separate peptides via a liquid chromatography (LC) system before analysis with MS [55] (Figure 4a). As well, there are diverse types of MS instruments depending on the ionization source and on the mass analyser (Figure 4b) [50]. Global proteome characterizations have been performed using tandem mass spectrometry (MS/MS). This allows a higher specificity of the mass spectrometer by coupling two analysers, using a collision cell and providing a quantitative protein expression profile. One of the quintessential proteomic techniques is ion mobility mass spectrometry (IM-MS), which involves the separation of ions using an electric field in the presence of a collision gas. IM-MS separates peptides from complex mixtures based on their mass/mobility trend line, increasing sensitivity and selectivity in peptide identification and quantification [92,93]. This approach enhances single-cell proteomic characterization when selectivity is increased by removing singly charged species, providing rapid sensitive analysis even at low concentrations [51]. Additionally, a proteomic analytical strategy is mass spectrometry imaging (MSI), which generates high-resolution chemical maps of complex samples such as biological tissues or materials surfaces without the need of labelling, and is widely applied in biomedical research [94].

On the other hand, protein microarray-based methods enable the identification of various molecules through affinity interactions with a panel of biomolecules immobilized onto a solid support. They consist of high-throughput matrixes that allow the study of specific protein characteristics (biochemical activity, interactions, and functions) in a miniaturized and large-scale manner with a minimum amount of sample [95]. Protein microarrays can be classified according to their format (planar or microsphere microarrays), their detection method (label-based and label-free microarrays), and their content, meaning the nature of the capture agent deposited on the surface (analytical, functional, and reversed-phase microarrays) [96] (Figure 4a). The main advantage of protein microarrays is the ability to generate high-content information with a limited amount of sample (such as complex and non-fractionated proteome mixtures, such as plasma, serum, or urine), which can be directly evaluated to determine differential protein profiles [97].

**Figure 5:**
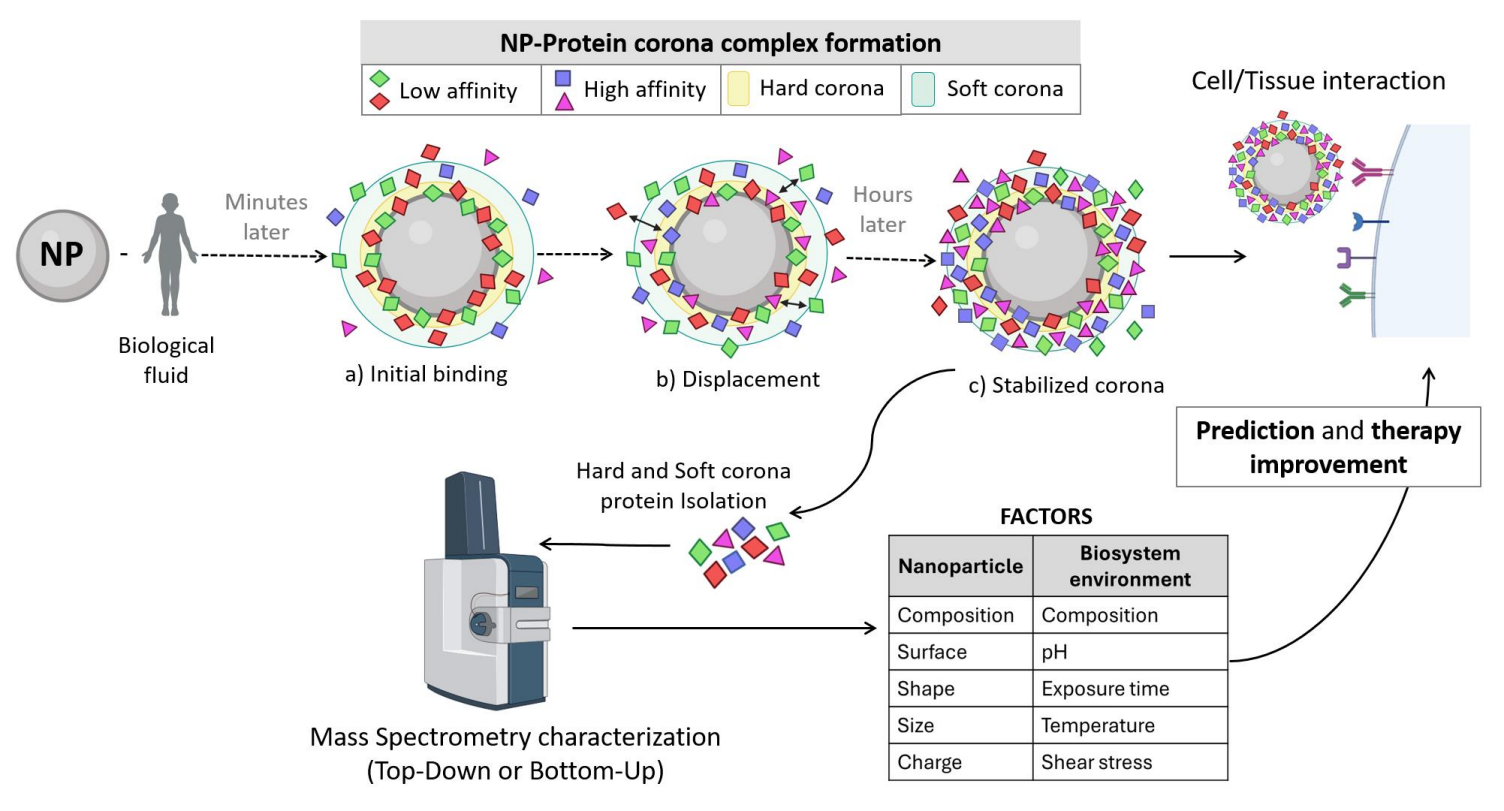
Illustrative diagram of protein corona formation when the nanoparticle is in contact with a biological fluid: (**a**) When the nanoparticles interact with the plasma, they become enveloped by low-affinity proteins present in high concentrations. (**b**) Low-affinity protein displacement by higher-affinity proteins. (**c**) The protein corona becomes stabilized hours later. The soft corona layer is formed by low-binding-affinity proteins, while the hard corona is composed by high-binding-affinity proteins. The characterization of these proteins (both hard and soft corona) reveals distinct factors to be considered for biomaterial functional improvements, such as for tumour targeting, diagnosis prediction, or immunotherapy.

With this variety in methods for proteomic approaches, it is essential to align them with the biological aspects related to biocompatibility. Specifically, it is important to identify which proteins are involved in biocompatibility mechanisms that are evaluated. As already seen, biomaterial toxicity and biofunctionality are influenced by local effects (Figure 2) and systemic response after the administration, integration, degradation, and possible accumulation [98]. To assess biocompatibility and guarantee patients’ safety—following ISO 10993-22:2017 [44]—it is essential for nanomaterials to undergo testing, not only for cytotoxicity but also for genotoxicity, immunotoxicity, systemic toxicity, and hemocompatibility. In this context, the interaction between proteins and nanomaterial surfaces is critical in the biological response as it mediates interactions with the cells and tissues [8,99]. Indeed, this inherent and spontaneous phenomenon of non-specific plasma protein coating on nanomaterial surfaces during the FBR process has been formally named the “protein corona”. It holds significant relevance in nanotechnology and nanomedicine [100,101] (Figure 5).

The exploration of protein corona formation (encompassing the adsorption, modification, and interaction of proteins with nanomaterial surfaces) has been a main research goal in the development of implantable devices during the last decades. Thus, there is a need for a systematic, high-throughput screening method to analyse this process since nanomaterials are exposed to mixtures of proteins upon implantation in the human body [102]. Gaining a deeper understanding of protein corona modifications and interactions will enhance biocompatibility and advance the development of drug delivery systems and other applications [8] (Figure 5).

In fact, protein corona formation is directly influenced by how the nanomaterial interacts with cells and tissues. This interaction is also affected by the material surface chemistry, roughness, size, and charge, among others [103,104]. Therefore, most of these factors are associated with the intrinsic properties of the material, which are critical for its biological compatibility and subsequent successful integration [38,105] (Figure 5). Surface chemical functionalization provides loading versatility and can be tailored for therapeutic drug immobilization, controlled intracellular release, or membrane mimicry [106]. Accordingly, the success of each targeted reconstructive strategy depends on the proper selection of the biomaterial along with the reaction of the creation of an adequate microenvironment [107].

Therefore, to further enhance our understanding of the dynamic interplay between proteins and solid surfaces [108,109], proteomic approaches are highly useful to profile the proteins expressed within a cell in real time and the protein corona arising in the nanomaterial [49,110]. For instance, profiling can be performed through MS, protein quantification using isobaric tags (e.g., tandem mass tag (TMT) [111,112]), or post-translational modification analysis. These technologies have been employed to establish connections between the proteins adsorbed on surfaces and subsequent biological responses [113,114]. However, this not only includes studying differential protein profiles, protein dynamics, and/or protein alterations, but also long-term monitoring to track time-dependent effects on evolution and immune tolerance [115,116].

### 3.1. Systemic Toxicity and Immunotoxicity

Systemic toxicity is one of the major risk factors associated with the use of medical devices. This issue can suggest the existence or pre-existence of immunotoxicity. In other words, it may alter the IS, resulting in either an overstated immune reaction or immunosuppression (which could generate more susceptibility to suffering recurrent infections) [117,118,119]. Considering the FBR process and acute inflammation process [30,33] (Figure 2), systemic immune monitoring during the early stages after the insertion is essential to avoid systemic infections or adverse effects that may reduce or cancel the treatment efficacy. In addition, the ability to control the structured placement of cells onto a substrate has recently gained a growing significance in tissue engineering. This controlled cell patterning will determine whether a biomaterial elicits an inflammatory response or rejection of the implant, even if it is immune-compatible within the receptor tissue [120,121].

In this regard, with biomaterials intended for direct contact with blood (such as vascular stents or nanoparticles), hemocompatibility plays a critical role, especially when considering the protein corona. Hemocompatibility involves assessing how the material interacts with all blood components (cellular and plasmatic biomolecules), including platelets and clotting factors [122]. The protein displacement that occurs in the protein corona before stabilization is directly related to dynamic changes in plasma [115]. Given the particular importance of the protein corona, it is important to consider synthetic and compatible nanomaterials to overcome potential complications.

Given this perspective, proteomic approaches analyse cellular proteins in real time, revealing cell status and protein corona formation on biomaterials [49,110]. Along with proteomic methodologies, MS and microarrays allow the detection, identification, and quantification of proteins and/or peptides in a huge diversity of samples (e.g., cell lysate, serum/plasma, etc.). To provide an overview of the applicability of these proteomics methods in the evaluation of immunotoxicity and systemic toxicity, some relevant examples are briefly described in this work.

As one of those proteomic studies, different highly sensitive protein microarrays have been designed to determine relative protein abundance in high-throughput and multiplex formats [123]. These microarrays have been employed for different purposes such as biomarker discovery (diagnostic and prognostic), potential vaccine development, and protein characterization, among others [96]. Below, we highlight the characteristics of the main microarrays that may be most relevant for studying immunotoxicity and systemic toxicity.

In the case of protein microarrays, multiple capture antibodies are printed onto a solid surface and exposed to a single protein lysate. When cells are infected by microorganisms (e.g., after transplantation), they can die, preventing the replication and spread of the pathogen. Cell death associated with the presence of PAMPs, or DAMPS, can stimulate the IR, leading to the recognition of specific antigens. These antigenic responses can be quantified by protein microarrays [124,125]. Even so, this approach requires highly specific targeted antibodies.

Reversed-phase protein arrays, on the other hand, are based on the simultaneous detection of a single protein, usually by antibodies, in multiple samples. This allows for an efficient quantification of tissue proteins and analysis of signalling cascades from very low amounts of sample (such as cell lysate) [123,126]. Furthermore, even phosphoproteins and other post-translational modifications can be detected by functional protein microarrays [123,127]. The disadvantage is that specificity may be compromised, because cross-reactivity may appear when a single detection probe/antibody is employed against a full content of proteins; for example those presented in a cell lysate, see [94]. By this proteomic technology, it is feasible to evaluate the activation [128], gain of function, or downregulation of proto-oncogenes [129]. In addition, essential protein interactions that are relevant to cell cycle progression or arrest can also be predicted by nucleic acid programmable protein array (NAPPA) [130].

Tissue microarrays allow the simultaneous proteome analysis of thousands of tissue samples on a single microscope slide [48]. Tissue microarrays can assess the distribution of targeted proteins in a variety of tissues, providing insight into the efficacy and toxicity to evaluate therapeutic effects. These arrays have been employed in many onco-proteomics studies, diagnostic test development, biomarker discovery, treatment monitoring, and the evaluation of histology-based laboratory tests (e.g., IHC and FISH). Additionally, tissue arrays can be used in clinical surveillance, covering different stages of disease progression within an organ, across multiple histological tumour types, or with frozen samples [94,131,132]. This approach is fast, high-throughput, and provides automated data analysis, thereby conferring a significant advantage in monitoring the host’s response to a biomaterial. However, it requires laborious construction, as tissue arrays are heterogeneous and poorly representative [48].

In addition to protein microarrays, MS-based technologies have been quickly developed in the last decade as they can also provide deep information on the molecular mechanisms involved in the IR [133]. This powerful technique offers the opportunity for the characterization of inter- and intracellular signalling pathways (quantitative proteomics) [108], an analysis of the abundance, modifications, and interactions between them, and the identification of novel biomarkers by establishing differential protein signals (de novo discovery) [123,134].

In the case of MALDI-MS (TOF and MSI), it allows the possibility of correlating the molecular information and the spatial and histological localization after MS measurement [94]. It is a label-free detection method that allows for a simultaneous multiplex analysis of several molecules in the same tissue sample and has been widely used in clinical proteomics [135,136,137]. Nowadays, imaging proteomics is a promising area in the clinic, mainly in spatial omics characterization.

Shotgun proteomics offers a hypothesis-free analysis by examining a broad range of peptides and proteins without targeting specific ones. Although it is not ideal for clinical routines due to complex sample preparation and the limited detection of low-abundance proteins [108], this approach enables a systematic assessment of immunotoxicity during discovery and validation phases. Additionally, when biocompatible materials degrade or break down, they may release byproducts with biological effects that can be analysed using MS.

LC-MS/MS plays an important role in liquid biopsy for predicting immunotherapy clinical outcomes and toxicity [138]. As a result of this potential proteomic application, Millet, A. et.al. used LC-ESI-MS/MS methodology to evaluate PD-1 blocking oncotherapies for reducing the administration dose and consequently, reducing the possible toxic effects [139]. However, the coupling of proteomics to the study of systemic toxicity offers a great opportunity for dose adjustments to reduce their adverse consequences.

### 3.2. Biofunctionality: Cell–Nanomaterial Interactions

Biocompatibility also requires the evaluation of the host–nanomaterial interactions, such as adaptiveness, biomimicry, tolerability, or biodegradation [103,140]. The rapid coating of nanomaterial surfaces by various extracellular molecules and the protein corona formation can modulate and influence the cell behaviour. These effects, or tissue responses, can include alterations in cell adhesion, spreading, differentiation, and proliferation, among other intracellular effects, as well as DNA damage or reactive oxygen species formation [141]. In addition, some nanomaterials must also demonstrate long-term stability within the tissue and the body [142]. Material stability is directly related to several biological and physiological aspects such as resistance to degradation, the maintenance of their structural integrity, and efficiency while allowing the integration of new tissues [143].

**Figure 6:**
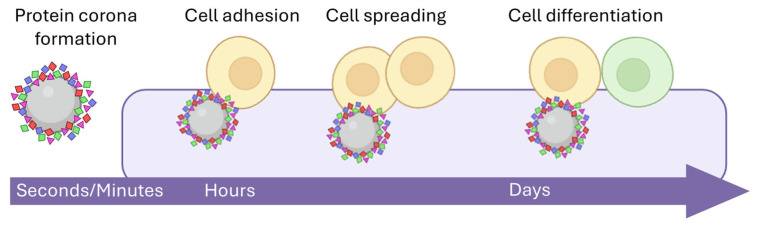
Cell–nanomaterial interactions: After protein corona formation, the complex interacts with specific cells and tissues. Firstly, attachment or adhesion is produced. As time goes by, the tissue responds, and the cells divide and differentiate. These interactions are an essential part of the study of biocompatibility. Within these interfaces, cell adhesion and spreading are evaluated, together with the biological compatibility ensuring their biosynthetic function.

Hence, it is highly relevant to control the cellular activity by tailoring the mechanical and biochemical characteristics of materials. These features can promote or avoid cell proliferation and attachment in the context of tissue engineering, cancer therapies, and nanomedicine [140,144]. All of these vital cellular dynamic processes have an influence on healing, regeneration, or even on embryonic development [144]. The cell should familiarize itself with the foreign material while maintaining its usual behaviour, avoiding uncontrolled proliferation and preventing tumorigenesis associated with the nanomaterial [145] (Figure 6).

#### 3.2.1. Cell Adhesion

Natural extracellular matrix proteins play an essential role in molecular interactions, particularly in cell adhesion; thus, biomaterials should mimic those matrix properties to allow cell growth and binding [146]. This is one of the aims of tissue engineering, to restore damage and facilitate tissue regeneration through peptide-based nanomaterials [147,148]. To study cell–nanomaterial interactions, it is necessary to identify the adsorbed proteins bound to the surface [99,149].

As outlined earlier, MS-based quantitative proteomics helps in understanding proteome changes, surface protein coating, protein distribution, and relative abundance. The quantitative evaluation of adsorbed plasma proteins by MS-based proteomics onto different surface materials helps to achieve better cell adhesion and growth in prosthesis. An example is silicate-based bioceramic surfaces for orthopaedic prosthesis instead of the conventional hydroxyapatite that had improved osteoblast adhesion [150].

Coating materials with bioactive molecules (e.g., chitosan, collagen, polyethylene glycol (PEG), or hydroxyapatite) enhance the biological activity and targeted protein adsorption, essential for cell colonization [151]. Functionalized nanomaterial surfaces, by binding short peptides, expand their novel and potential applications [104]. One of the representative examples is the short peptide (sequence arginine-glycine-aspartic acid (RGD)—integrin), which is commonly used in nanoparticle surface binding for improving targeting [152,153]. Nanoscale modifications on these RGD sequences led to significantly decreased cell adhesion and migration, and can potentially be used for metastasis inhibition [154]. Lü X. et al. quantitatively measured serum adsorbed proteins by LC-ESI-MS/MS, showing that chitosan +/− combined with collagen films increased cell adhesion, due to the high amount of proteins with RGD and LDV (leucine-aspartic-valine) motifs as adhesion regions [155].

Cell–nanomaterial interactions can be enhanced by deciphering specific protein profiles and chemical moieties of the nanomaterial surface [156]. Material surface and cell interactions affect inflammation and cellular adhesion differently [157]. Then, novel protein networks are unravelled by measuring up/downregulated proteins like Rictor/mTORC2 involved in cell adhesion regulation [158]. Furthermore, the identification of adhesion molecules, during protein coating, by LC-ESI-Orbitrap-MS/MS (such as fibrinogen, albumin, and complement C3 or C5) has contributed to addressing local reactions to silicone driven by breast implants [159].

Bearing in mind that cell adhesion is crucial in orthopaedic implants and is influenced by protein adsorption, increasing adherence can accelerate bone regeneration. The characterization of biomaterials by LC-ESI-TOF-MS/MS has revealed key nanotextures for enhancing mineralization and bone healing. For instance, magnesium-doped materials promote cell adhesion and anti-inflammatory effects [160], TGF-β3 stimulates osteoblast metabolism [161], and gelatine coating surfaces mimic collagen I and have a better IR [162].

On the other hand, other approaches try to prevent cell adhesion, as happens during thrombogenic effects after implantation [163]. With this objective, developing an antithrombogenic surface that reduces platelet coagulation while maintaining cellular compatibility is a challenge. Some strategies include changing ceramics’ crystallographic orientation to alter platelet activation [164], avoiding the coagulation cascade thanks to nitinol surface treatment with phosphate and calcium ions [165], or using a custodial solution in acellular liver scaffolds to enhance hemocompatibility [166]. The LC-ESI-MS/MS method, together with DIA analyses of patients’ coagulation profiles, revealed differences in coagulation pathways during haemodialysis therapy, depending on the material used. Identifying proteins adsorbed onto the dialysis membrane is key to understanding these events and engineering new membrane materials, such as modified cellulose or poly-sulfone membranes, compatible with patients’ plasma [167].

Assessing biocompatibility also involves considering implant colonization by external microorganisms, and proteomics may uncover the possibility of pathogen adhesion, which could be monitored by LC-MS/MS or protein microarray approaches. LC-MS/MS has been employed to study saliva and plasma proteomic profiles post-implantation of acid-etching titanium–zirconium implants, which enhance biological effects and promote bacterial adhesion [168]. Meanwhile, proteomic polymer arrays had engineered materials resistant to bacterial attachment [169,170]. Another antimicrobial approach characterized by MS is the functionalization of gold nanoparticles with polyoxometalate coronas for combating antibiotic resistance [171].

#### 3.2.2. Cell Spreading

As described in previous sections, microenvironment signals depend on multiple factors, such as the extracellular matrix (ECM), physical structure, chemical composition including soluble factors (chemokines, cytokines, growth factors, etc.), or selective cell adhesion–spreading equilibrium, which ultimately determine cell behaviour [144]. Nanoparticles can mediate in this microenvironment signalling, regulating protein expression and subsequent effects like specific cell recruitment, vascularization, or remodelling during tissue formation. For instance, proangiogenic nanomaterials such as nano zinc oxide, which promotes the migration and formation of new capillaries, are a promising strategy in tissue engineering [172].

ECM plasticity regulates mesenchymal stem cell (MSC) spreading, which is conditioned by exosomes, influencing their behaviour and on neighbouring cells. MSC secretome characterization contributes to designing materials for sustained secretion-enhancing paracrine benefits [173]. This has made it possible to develop strategies for improving oral bone regeneration and skeletal muscle implant healing [174,175,176]. For example, immobilizing exosomes in titanium surfaces promoting MSC adhesion, spreading, and proliferation [177] or coupling soluble ligand gradients to ECM materials are promising techniques for optimizing cell migration in scaffolds for skin regeneration [178,179].

For reaching this characterization, conventional techniques (such as immunohistochemistry or Western blot) can evaluate the expression level of individual or low-number proteins. Conversely, the evaluation of protein expression levels (differential protein profiles) of high numbers of proteins could be deciphered by high-throughput proteomics methods such as MS and protein microarrays [180,181]. As an example, promising pulmonary injury regeneration, by the inhalation of spheroid exosome and secretome (using a nebulizer for direct delivery to the lungs), has been successfully characterized by LC-ESI-Orbitrap-MS/MS and DDA acquisition methods [182]. In urological diseases, the same MSC secretome components characterization allowed the identification of key components, such as pigment epithelium-derived factor, for addressing cellular therapy drawbacks like immunoreactivity [183].

Microenvironment signalling and soluble factors are pivotal in regulating spreading. A comprehensive proteomic analysis of soluble factors in response to nanomaterial exposure contributes to regulating, not only cell spreading, but also overall tissue functionality [184]. Some recent illustrative findings are outlined below.

Firstly, tissue regeneration aims to restore functionality, demanding effective vascularization for healing—a process characterized by a high rate of cellular spreading. Angiogenic agents in liver scaffold failures have been identified and interconnected by LC-ESI-Orbitrap-MS/MS analysis over time. This work, led Guo B., et al., suggests that 14 days of in vitro maturation is necessary for specific angiogenic co-expression and the consequent development of a mature vascular network, which may increase host efficiency [185]. Recently, Guo B.’s group used this basis to perform the first orthotopic transplantation of functional bioengineered livers prolonging survival in rats undergoing complete hepatectomy [186]. Moreover, a vitronectin protein coating has been suggested by protein microarrays as a vascularization promoter in porous polyethylene implants for auricular and facial contour reconstruction [187].

A favourable microenvironment is a challenge for efficient nerve regeneration. The protein profiles (by LC-ESI-TOF-MS/MS) of Schwann cells on poly (caprolactone) fibrous scaffolds functionalized with glycosaminoglycan were compared on days 3 and 7. The results indicate that day 7 is the only point with a robust proteomic comparison. These findings suggest that GAG-functionalized scaffolds recreate natural Schwann cell basal lamina, boosting proliferation, differentiation, and migration into the healing nerve [188].

Continuing with tissue regeneration, advances in protein expression profiling by MS/MS highlight promising properties in bone marrow mesenchymal cell-derived ECM in peripheral nerve repair by recruiting endogenous cells and nerve regeneration cell type-derived ECM [189]. Another significant development in tissue engineering is the regulation of synthetic hydrogels through external stimuli in dynamic scaffolds. LC-ESI-MS/MS characterized disulfide photo-remodelling hydrogels for in situ cell encapsulation and matrix remodelling, enabling controlled tissue formation [190].

In cell recruitment strategies, which involve cell migration and spreading, LC-ESI-MS/MS and DDA comparative analysis have revealed that degradable polar hydrophobic ionic polyurethane film induces monocyte-derived macrophage secretion. This promotes an anti-inflammatory macrophage phenotype and consequently minimizes fibrosis in cardiac implants [191].

The up- and downregulation of specific proteins depending on the nanomaterial surface is a key point for addressing cell migration. For example, hyaluronan, an essential matrix component, when deregulated, can lead to different pathologies such as cancer or tissue fibrosis [192]. Tumour invasion and migration in vitro studies employ early protein deposition on hyaluronic acid-based hydrogels to support cell spreading [193,194]. High-performance liquid chromatography (HPLC) analysis has discovered how adhesive hydrogels mimic ECM properties. This benefits corneal defect regeneration by downregulating proteins involved in cell death processes and upregulating others related to migration, adhesion, and healing [195,196].

Moreover, the MALDI-TOF-MS technique has been used to confirm peptide gradient concentration profiles as part of a strategy for guiding directional cell migration and tissue regeneration. For instance, RGD and YIGSR peptides increased the migration velocity and selectivity of Schwann cells in nerve repair [197]. In another report, the MALDI-ICR-MS technique was used to reveal the main peptides in snail slime that induced organic gold nanoparticle formation, which upregulates the urokinase-type plasminogen activator receptor in human keratinocyte adhesion, spreading, and epidermal healing [198].

#### 3.2.3. Cell Biosynthetic Function

In the domain of regenerative medicine and tissue engineering, it is necessary to develop biomaterials capable of mimicking the biosynthetic function of a specific organ or tissue (such as dental, kidney, or corneal implants and orthopaedic materials) [199]. Biomimetic materials have become increasingly popular in those fields. They can replicate the natural microenvironment and offer cells a broad spectrum of biochemical and biophysical properties that simulate the in vivo extracellular matrix. Moreover, these materials can be tailored to have mechanical adaptability, ensuring optimal support for cell growth, microstructure interconnectivity, and inherent bioactivity. These properties further enhance their suitability for designing living implants with specific regenerative applications [200,201].

Alternatively, bioactive materials elicit specific cell responses. Their bioactivity range extends from the general and already mentioned cell adhesion, spreading, or proliferation to more specific tasks, such as immune modulation for cancer immunotherapy or protein aggregation inhibition in neurodegenerative diseases [202]. For example, bioactive materials are used for the biomimetic mineralization of dentin in the replacement of damaged dental tissues [203].

Due to the necessity of a bioactive surface that enhances tissue response, Zuanazzi et al. studied the formation of a specific protein layer strategy. They investigated how titanium surface modifications influence salivary pellicle composition to enhance tissue response. Using LC-ESI-MS/MS, they analysed the salivary proteome and found that rough surfaces formed a more complex protein profile. The study identified key salivary proteins adsorbed on titanium surfaces that are involved in IR, adhesion, and biomineralization [204]. In this sense, the functionalization of nanoparticles to increase their bioactivity in certain tissues is also increasing, such as gold and silver nanoparticles for skin engineering [205].

The LC-ESI-MS/MS approach was also used to evaluate protein deposition on different calcium-enriched titanium surfaces. Romero-Gavilan et al. concluded that osteogenic and inflammatory responses vary with the calcium coating dose. They also observed that higher calcium affinity for anti-clotting proteins increases the coagulation potential of these materials [206].

Nephron, the functional units of the kidney, are composed of tubular structures. Recent advances in kidney tissue engineering have focused on developing biosynthetic tubules using perfusable microfluidic proximal tubule chips and scaffolds to support engineered kidney units [207]. In this regard, the LC-ESI-Q-MS/MS method has been employed to measure the expression of renal transporter peptides released by tubular cells, aiming to design novel anti-fibrotic drugs to prevent progressive kidney function loss [208].

### 3.3. Systematic Proteomics Characterization of Nanomedicines

Nanomedicine involves the use of nanocarriers for drug delivery in multiple pathologies and other applications integrating a wide variety of scientific disciplines (biology, chemistry, physics, and technology). Over the past decades, nanomedicine has emerged as a revolutionary field to address different medical challenges such as drug delivery, diagnostics, imaging, or tissue engineering [209]. The interplay between proteomics and nanomedicine paves the way for personalized medical approaches by enabling precise drug delivery and tailored therapies based on a deep understanding of protein functions and interactions [210]. For instance, UPLC-MS/MS was performed to evaluate niclosamide- encapsulated lipid particles as efficient drug-delivery systems for reversing pulmonary fibrosis [211].

The protein corona identification by proteomics expands the targeting strategies for new therapeutic agent development and novel biomolecular pattern identification for disease diagnosis [100,116]. For example, (MS)-based top-down proteomics has contributed to the characterization of hundreds of proteoforms in the protein corona of polystyrene nanoparticles, revealing new protein biomarkers [212].

Concerning personalized medicine, biomimetic nanovaccines are being developed for treating cancer or infectious diseases as an alternative to antibiotics or tumour resistance [213,214]. For instance, LC-ESI-TOF-MS/MS has recently contributed to a promising proposal for an oncotherapy based on nanovaccines induced by chemotherapy that stimulates the IS and reduces the cytotoxicity [215].

Another major strategy in nanomedicine is the use of exosomes, which provide a promising nanotherapeutic strategy for multiple diseases as drug delivery systems [216]. Proteomic analysis has promoted the presentation of exosome-based nanocarriers as anticancer theragnostic due to their biocompatibility, stability, immunogenicity, and high loading capacity [217,218]. LC-ESI-Q-MS/MS studies and the DDA acquisition method reveal a platform to treat cartilage degeneration during osteoarthritis that consists of chondrocyte-targeting exosomes encapsulating functional siRNAs [219].

Exosome application has significant impact on early diagnosis for neurological diseases. LC-ESI-MS/MS has been used to compare exosomal proteins, revealing a list of glioblastoma biomarkers in order to create a non-invasive diagnostic technique [220]. Quadrupole high-resolution mass analysis has characterized exosome-related proteins that may reflect the physiological status of diseases, which could speed up the diagnosis of central nervous system neoplasms [221]. Similarly, a TMT-labelled MS/MS approach has identified exosomal fibulin-1 exosomes as a potential biomarker for diagnosing mild cognitive impairment of Alzheimer’s disease [222]. Although great advances have been made in proteomics exploration of exosomes [223], this field continues to expand and offer novel insights into their cargo selection, biogenesis, and potential as biomarkers [218].

Additionally, proteomics offers an outstanding opportunity to study intracellular processes in detail, which were most previously studied only by genomics. One of those proteomics methodologies is based on NAPPA technology, which, combined with cell-free nanobiocrystallography, offers an integrated approach to study specific protein cascades and interactions. These protein microarrays could overcome current challenges in medical diagnosis and therapy related to T-lymphocyte transformation to lymphoma [224].

Regarding drug discovery, proteomics contributes significantly to target identification and response monitoring. A leading-edge technology in this field is MS-based single-cell proteomics, whose potential is growing in three fields: cell annotation (to study different profiles of cell subpopulations within a sample), developmental trajectories (intermediate cellular states over time), and spatial mapping (intracellular interactions to measure their original spatial arrangement) [225]. Regarding drug resistance studies, label-free bottom-up shotgun proteomics has recently revealed that p53 facilitates drug resistance in drug-sensitive cells through both short- and long-distance communication [226].

### 3.4. Chemoproteomics

Recently, related to target identification and response monitoring, proteomics has joined with chemical biology into a powerful emerging approach called chemoproteomics. Chemoproteomics involves the systematic identification and characterization of protein–ligand interactions through selective chemical probes and MS methods [227]. This approach is employed to study protein interactions, modifications, and subsequent cellular responses, including IR monitoring. Consequently, chemoproteomics enables the identification of key protein–nanomaterial interactions, the assessment of their adverse effects, the advancement of targeted drug discovery, and a more profound understanding of the mechanisms of action of compounds with potential therapeutic applications [228,229,230,231,232].

For protein identification, chemoproteomics encompasses affinity-based proteomics and activity-based protein profiling (ABPP) methodologies. ABPP is an advanced technique for characterizing specific protein targets of bioactive molecules, such as drugs or biomaterials, providing insights into potential toxicity mechanisms [227,233,234]. For instance, Li et al. investigated the anti-inflammatory effects of dihydrocaffeic acid in acute pneumonia by ABPP and LC-ESI-MS/MS. This study revealed that this acid alleviates inflammation by binding to transaldolase 1 and modulating the PERK-NF-κB signalling pathway, providing a potential therapeutic approach for treating acute pneumonia and for inflammation-related disorders [235]. Similarly, chemoproteomics approaches have recently been introduced as an effective strategy to directly identify protein targets of antimalarial compounds [236]. For example, Gao et al. applied an ABPP-based target profiling strategy to demonstrate Celastrol’s mechanism in disrupting spermidine and protein synthesis in the *Plasmodium falciparum* parasite, revealing its potential for developing biomaterials with antimalarial properties [237]. Another example is how chemical proteomics supports the discovery of the human kinome, thereby aiding in the development of therapeutic drugs for kinase biology regulation [232].

Continuing biocompatibility studies at the molecular level, nanomaterials can induce post-translational modifications (PTMs) (such as oxidation, phosphorylation, glycosylation, and ubiquitination) that influence cellular behaviour. PTM characterization enhances drug discovery and advances the development of new therapies [238]. It has been reported that the impact of oxidative stress on titanium dioxide dental implants for bone regeneration can influence the success of biomaterial implantation and longevity [239]. Additionally, Pillai et al. developed a single-cell chemical proteomic platform to identify metastatic activity signatures in breast cancer, showing how this technology can profile cellular behaviours at a highly granular level. Although their research has primarily focused on cancer metastasis, their studies highlight the potential of chemoproteomics in uncovering subtle cellular changes in response to biomaterials, particularly in the context of immune interactions and the development of immune-related complications [240]. Within this perspective, the chemical study of nanoparticle coatings is crucial for differentiating their pharmacological properties [116].

Leveraging high-throughput chemoproteomic approaches, a recently established method called the proteome integral solubility alteration (PISA) assay can study various samples or conditions simultaneously by thermal proteome profiling TMT labelling followed by LC-ESI-MS/MS and HPLC analysis. The PISA assay reduces sample consumption, analysis time, and experimental errors, increasing the screening scale of protein stability and solubility altered by various agents [241].

Moreover, the combination of chemoproteomics and metabolomics provides a holistic view of cellular responses. This integrated approach helps in deciphering how biomaterials influence metabolic pathways, oxidative stress, and inflammatory responses, contributing to biocompatibility evaluation. The integration of metabolomics with chemoproteomics and PTMs provides insights into drug–protein interactions, revealing drugs’ mechanisms of action (such as rapamycin, a compound used to prevent rejection in transplants) and predicting their potential side effects [242].

Together, these studies illustrate how chemoproteomics is a powerful tool for gaining insight into complex immune and cellular dynamics involved in materials biocompatibility testing and improvement.

## 4. Conclusions and Perspectives

Currently, health systems (https://www.who.int/health-topics, accessed on 27 March 2025) and One Health (https://www.onehealthinitiative.com, accessed on 27 March 2025) require the development of novel medical devices that take advantage of multiple and different characteristics of biomaterials. The recent and novel bioactive materials have faced clinical challenges and accelerated advances in fields such as pathology, dentistry, chemistry, molecular biology, and tissue engineering. However, to ensure biocompatibility and reliability, host–material interactions, which depend on multiple physical and chemical factors, must be rigorously tested for safety and toxicity in order to bring these nanomaterials from the laboratory to the clinic (“from the bench to the bedside”) [2,243].

Nowadays, some of the nanomaterial challenges are selecting high-performance materials, updating fabrication and processing techniques, strengthening the physical/chemical cross-talk, and optimizing their biostability. Biomaterial applications are also expanding, especially in regenerative medicine and engineering. Similarly, they have new issues to address such as specific target applications, drug and bioactive molecule delivery, systemic in vivo screenings, IR prediction and modulation, or microenvironment replication [244,245,246,247].

In vitro techniques have proved to be highly valuable for biomaterials evaluation, particularly in toxicology, by assessing cell viability, adhesion, proliferation, and biosynthetic activity [58] and other specific tissue hazards. Despite this, they are unable to address certain factors such as defined dose, final risk after implantation, or degradation [117]. Therefore, combining in vitro and in vivo techniques is required for integrated biomaterials testing. The expansion of in vitro testing methodologies is a way to customize biomaterials for specific in vivo applications, with both approaches being pivotal in the overall evaluation of biomaterials.

Although in vitro classic techniques are quite useful, they still present some limitations, such as false positive results, that raise some uncertainties about their applicability in therapeutic nanomaterials testing [248]. The lack of specificity in classic methods prevents them from deciphering cell damage origin or the exact cellular response, as well as revealing what occurs to the biomaterial’s structure, composition, binding, and behaviour.

At this stage, proteomics plays a crucial role offering more sensitive and precise screening and expanding the scope for exploration beyond the reach of traditional methodologies. In this regard, proteomics enables the characterization of proteins, which can be correlated with cell viability while also offering information about the material and cellular reaction, and the interface between them. However, it is important to underscore that proteomics does not supplant the necessity of conventional techniques for assessing critical biomaterial parameters such as cytotoxicity but adds an extra dimension to biocompatibility testing. High-throughput methods, such as microarrays and MS techniques, enhance the development and validation of biomaterials, increasing their capabilities. Therefore, high-throughput and high-content screening techniques are compatible and complementary to conventional viability and toxicity studies [244]. For instance, metabolic assays, such as the MTT assay, do not present high sensitivity, possibly necessitating specific metabolic pathway characterization, involving labelling induced proteins that serve as indicators of certain metabolic rates [249,250,251]. Functional proteomics tools, like protein microarrays, can identify different mutations or biomaterial effects on the cell cycle by discovering new cell signalling checkpoints [252,253]. Otherwise, MS could also increase knowledge of membrane interactions or contribute to the early detection of permeability loss when exposed to toxic compounds [254,255].

The biological response between the host and biomaterial is continuously evolving, and requires systematic and deep evaluation. Regarding interactions, MS characterization accelerates understanding and combined with MS imaging, spatial molecular information can also be explored [256]. For example, these techniques together have an enormous potential in the prevention, characterization, and evaluation of colonizing pathogens, especially in the material implantation phase and in wound healing applications.

Nevertheless, it is important to highlight an MS disadvantage in peptide identification and quantification: the false discovery rate (FDR), whose typical threshold is 1% [257]. Multiple strategies are being developed to address the accuracy of protein identification. One approach is targeted MS methods such as SRM or PRM, which provide precise protein quantification [258], or chemoproteomics, which includes PISA assays [241]. These strategies not only overcome some classical technique limitations but also offers target identification for reducing the FDR. Another approach for reducing the FDR is the target-decoy strategy, which controls the FDR during MS analysis by reversing or randomizing the protein sequences from the real database [259]. The FDR issue also concerns expression proteomics, which reveals real protein functional changes and is also influenced by protein corona modifications when in contact with biomaterials [260]. Although the FDR affects to protein corona identification, this manuscript provides evidence of proteomics’ value and versatility in the fields of nanomedicine and biomaterials.

By taking it a step further, the proteomic identification of specific compound targets can also be measured by incorporating a temporal dimension and studying the process over a dynamic range, as demonstrated by chemoproteomics and PISA assays. Although the introduction of chemoproteomics in biocompatibility assessment is a transformative advance in biomaterial medicine, its immediate future lies in its integration with machine learning and artificial intelligence-driven data analysis, empowering predictive models of biomaterial interactions [229,261].

Given chemoproteomics’ wide applications and rapid improvements, these proteomics approaches would allow faster improvement in the design and evaluation of new nanomedicines and new mechanisms, the exploration of new combinations of drugs, and the identification of possible mechanisms of resistance. Within this scope, new standardized protocols are emerging to analyse multiple samples, conditions, and compounds on an extensive scale. Some of the most recent proteomic approaches include thermal proteome profiling and PISA assays for drug target identification [262] or MS-based thermal stability assays for identifying protein–ligand interactions [263].

In summary, large-scale proteomic advances supply a powerful set of tools that, coupled with the rapid evolution of biomaterials, complement the traditional techniques to expand their evaluation and potential to improve society’s quality of life.

## Figures and Tables

**Table 1:** Viability evaluation techniques, with examples of some reagents used and biomaterial applications as well as recent limitations.

	Technique and Reagent	Applications for Biomaterials	Limitations
Dye exclusion assays	Selective penetration of dyes in dead or damage cells [64,65]. Trypan blue, neutral red, eosin, Congo red, and erythrosine B	Dicalcium and tricalcium silicate with hydroxyapatite endodontic cement on human lymphocytes [66]. ZnO nanoparticles for antimicrobial activity, targeted cancer therapy, drug delivery, and tumour detection [63].	Limited accuracy. Toxic to mammalian cells. Counting errors (inadequate cellular dispersion, incorrect dilution, air bubbles, inter-user variations, no difference between healthy and viable cells that have lost their functionality) [62,67,68].
Fluorescence assays	Fluorescent probes distinguish living and dead cells based on membrane permeability [69]. Fluorescein diacetate (FDA), propidium iodide (IP), Hoechst 33342, ethidium bromide, and SytoxGreen	Distinguish infiltrating cells from implanted mesenchymal stem cells in cartilage defects studies [70].	Signal overlapping when distinguishing multiple targets simultaneously. Non-specific bindings. Quenching. Fluorescence loss or photobleaching.
Flow cytometry approaches	Detect scattered and fluorescent signals for evaluating single cell characteristics (size, shape, granularity, viability, cell cycle analysis, etc.) [71,72]. APC, FITC, PE, IP, and annexin V	Targeted nanocarriers for prostate cancer cell reduction [73]. Magnesium-based medical implants [74].	Requires cells in suspension. Decreased detection sensitivity for viable cells at low sample concentrations. Spectral overlapping of some fluorochromes.
Metabolic assays	Assess cell metabolic activity using biochemical markers, indirectly determining cell viability [64,75,76,77,78]. Tetrazolium salts (MTT, XTT and WST-1), resazurin salts (Almar blue), fluorescein diacetate	Luminescent CaS nanoparticles for cancer hyperthermia treatment [79]. AlSb nanocrystals for retinal stimulation to rescue vision [80]. Gold nanoparticles as nano-immunotherapy carriers [81].	Inconsistent optical density correlations. Inability to distinguish dividing from quiescent cells; over-estimation of cell count [82,83].
Membrane integrity assays	Measure cells’ exclusion capacity for impermeable molecules, often through enzyme release or leakage assays [64,84,85]. Lactate dehydrogenase (LDH), annexin V, and aF2N12S probes	Necrotic status prediction in hernias [86].	Limited sensitivity (not able to detect early cell death or to differentiate between apoptosis and necroptosis). False positives due to membrane regeneration or interference with medium products or proper material [87,88].

## Abbreviations

The following abbreviations are used in this manuscript:

IS	Immune System
IR	Immune Response
PAMPs	Pathogen-associated Molecular Patterns
DAMPs	Damage-associated Molecular Patterns
TLR	Toll-like Receptors
FBR	Foreign Body Response
ISO	International Organization for Standardization
MS	Mass Spectrometry
DDA	Data-dependent Acquisition
DIA	Data-independent Acquisition
PRM	Parallel Reaction Monitoring
SRM	Selected Reaction Monitoring
LC	Liquid Chromatography
MALDI	Matrix-assisted Laser Desorption/Ionization
ESI	Electrospray Ionization
TOF	Time Of Flight
Q	Quadrupole
ICR	Ion Cyclotron Resonance
MSI	Mass Spectrometry Imaging
IM-MS	Ion Mobility Mass Spectrometry
TMT	Tandem Mass Tag
NAPPA	Nucleic Acid Programmable Protein Array
PEG	Polyethylene glycol
RGD	Arginine-glycine-aspartic acid
ECM	Extracellular matrix
MSC	Mesenchymal Stem Cells
HPLC	High-performance liquid chromatography
ABPP	Activity-based Protein Profiling
PTM	Post-translational Modifications
PISA	Proteome Integral Solubility Alteration
FDR	False Discovery Rate
